# Intraspecific variation in aerobic and anaerobic locomotion: gilthead sea bream (*Sparus aurata*) and Trinidadian guppy (*Poecilia reticulata*) do not exhibit a trade-off between maximum sustained swimming speed and minimum cost of transport

**DOI:** 10.3389/fphys.2015.00043

**Published:** 2015-02-17

**Authors:** Jon C. Svendsen, Bjørn Tirsgaard, Gerardo A. Cordero, John F. Steffensen

**Affiliations:** ^1^Molecular Eco-physiology, Interdisciplinary Center of Marine and Environmental Research, University of PortoPorto, Portugal; ^2^Fisheries and Maritime MuseumEsbjerg, Denmark; ^3^Marine Biological Section, Biological Institute, University of CopenhagenHelsingør, Denmark; ^4^Ecology, Evolution, and Organismal Biology, Iowa State UniversityAmes, IA, USA

**Keywords:** aerobic metabolic scope, anaerobic capacity, burst swimming, excess post exercise oxygen consumption, intraspecific variation and trade-off, locomotion, maximum sustained swimming speed, minimum cost of transport

## Abstract

Intraspecific variation and trade-off in aerobic and anaerobic traits remain poorly understood in aquatic locomotion. Using gilthead sea bream (*Sparus aurata*) and Trinidadian guppy (*Poecilia reticulata*), both axial swimmers, this study tested four hypotheses: (1) gait transition from steady to unsteady (i.e., burst-assisted) swimming is associated with anaerobic metabolism evidenced as excess post exercise oxygen consumption (EPOC); (2) variation in swimming performance (critical swimming speed; *U*_crit_) correlates with metabolic scope (MS) or anaerobic capacity (i.e., maximum EPOC); (3) there is a trade-off between maximum sustained swimming speed (*U*_sus_) and minimum cost of transport (COT_min_); and (4) variation in *U*_sus_ correlates positively with optimum swimming speed (*U*_opt_; i.e., the speed that minimizes energy expenditure per unit of distance traveled). Data collection involved swimming respirometry and video analysis. Results showed that anaerobic swimming costs (i.e., EPOC) increase linearly with the number of bursts in *S. aurata*, with each burst corresponding to 0.53 mg O_2_ kg^−1^. Data are consistent with a previous study on striped surfperch (*Embiotoca lateralis*), a labriform swimmer, suggesting that the metabolic cost of burst swimming is similar across various types of locomotion. There was no correlation between *U*_crit_ and MS or anaerobic capacity in *S. aurata* indicating that other factors, including morphological or biomechanical traits, influenced *U*_crit_. We found no evidence of a trade-off between *U*_sus_ and COT_min_. In fact, data revealed significant negative correlations between *U*_sus_ and COT_min_, suggesting that individuals with high *U*_sus_ also exhibit low COT_min_. Finally, there were positive correlations between *U*_sus_ and *U*_opt_. Our study demonstrates the energetic importance of anaerobic metabolism during unsteady swimming, and provides intraspecific evidence that superior maximum sustained swimming speed is associated with superior swimming economy and optimum speed.

## Introduction

Variation in locomotor performance and metabolism is linked to fitness, because both traits are often coupled with important behaviors such as predator evasion, prey capture, reproduction, migration, and dominance (Clobert et al., 2000; Walker et al., 2005; Langerhans, 2009a; Leis et al., 2009; Eliason et al., 2011; Seebacher et al., 2013; Wilson et al., 2013; Burnett et al., 2014; Killen et al., 2014; Scantlebury et al., 2014). Intraspecific variation in locomotor performance and metabolism is repeatable across time and environments (Chappell and Odell, 2004; Claireaux et al., 2005, 2007; Oufiero and Garland, 2009; Norin and Malte, 2011, 2012; Careau et al., 2014) and may be heritable and/or trans-generational (Rønning et al., 2007; Dalziel et al., 2011, 2012; Dalziel and Schulte, 2012; Gore and Burggren, 2012; McKenzie et al., 2013; Mattila and Hanski, 2014), indicating that traits related to locomotor performance and metabolism are subjected to natural selection and could evolve over time.

Performance trade-offs are central to understanding the vast phenotypic variation found among species, populations, and individuals. Trade-offs may occur when two antagonistic traits cannot be optimized simultaneously, because the two traits pose conflicting demands on the same design feature (Damme et al., 2002). Consequently, excellence in one trait will come at the cost of performance in the other trait (Vanhooydonck et al., 2014). Hence, an organism may specialize in one trait at the cost of the other, in which case a trade-off may cause phenotypic differentiation (DeWitt and Scheiner, 2004; Konuma and Chiba, 2007; Herrel et al., 2009). Alternatively, the conflicting demands may result in organisms performing sub-optimally for both traits and therefore, constrain evolution (Lewontin, 1978; Arnold, 1992). In fish, there is evidence of a trade-off between endurance capacity and sprint speed (Langerhans, 2009b; Oufiero et al., 2011); however, the trade-off is not ubiquitous at the whole-organism level (Wilson et al., 2002; Vanhooydonck et al., 2014; Fu et al., 2015).

Levels of swimming exercise in fishes have been divided into three categories on the basis of the time a given speed can be maintained before the onset of fatigue (Beamish, 1978): sustained (more than 200 min), prolonged (20 s to 200 min) and burst swimming (less than 20 s). In many teleosts, the segmented myotomal musculature is distinctively divided into red oxidative (slow-twitch) muscles and white glycolytic (fast-twitch) muscles. Red muscles are powered by oxidative phosphorylation, whereas white muscles are largely powered by anaerobic utilization of phosphocreatine, ATP and glycogen. At sustainable swimming speeds, the red musculature is powering propulsion, whereas white musculature is increasingly recruited for propulsion at faster speeds. Employing white musculature for burst-assisted swimming typically involves significant physiological perturbations including decreasing levels of intracellular pH (Lurman et al., 2007) and muscle glycogen (Peake and Farrell, 2004), and increasing levels of lactate (Martínez et al., 2004; Peake and Farrell, 2004) and inorganic phosphate (Lurman et al., 2007) indicating a non-steady state and partial reliance on anaerobic metabolism. While metabolic locomotor cost during sustained swimming has received considerable attention (Brett, 1964; Steinhausen et al., 2005; Ohlberger et al., 2006; Svendsen et al., 2013), the metabolic cost during non-steady burst swimming remains poorly understood. Metabolic locomotor cost during sustained swimming can be estimated using measurements of instantaneous oxygen consumption rates (exercise *M*O_2_), whereas metabolic cost during unsustainable swimming can be estimated by combining exercise *M*O_2_ with excess post exercise oxygen consumption (EPOC). The presence of EPOC is considered evidence of anaerobic activity in intact fish (Beamish, 1978), with many of the physiological perturbations related to anaerobic metabolism cleared during the period associated with EPOC (Peake and Farrell, 2004). In striped surf perch (*Embiotoca lateralis*), a labriform swimmer, there is a linear relationship between the number of bursts and EPOC, with EPOC constituting 25% of the total swimming costs (total *M*O_2_; i.e., exercise *M*O_2_ and EPOC combined) on average (Svendsen et al., 2010). In contrast, the metabolic cost of burst swimming in axial swimmers is largely unknown (Puckett and Dill, 1984; Farrell, 2007).

Standard metabolic rate (*M*O_2stand_) is a basic maintenance requirement measured as the minimum rate of oxygen consumption of postprandial unstressed animals at rest, below which physiological function is impaired. Long-term energy demands for swimming, food acquisition and treatment, regulation owing to environmental perturbations, and reproduction are additional to standard metabolism. These demands are met within the range set by the maximum metabolic rate (*M*O_2max_) (Priede, 1985). The difference between *M*O_2stand_ and *M*O_2max_ is termed the metabolic scope (MS). Because MS is strongly influenced by environmental variables, including temperature and oxygen availability, MS is predicted to be a major physiological variable in relation to climate change and aquatic hypoxia (Claireaux and Lefrançois, 2007; Chabot and Claireaux, 2008; Guderley and Pörtner, 2010; Pörtner, 2010; Pörtner and Peck, 2010; Di Santo, 2015). Nevertheless, intraspecific relationships between MS and other important physiological traits have rarely been explored in detail. MS and swimming performance correlate positively in intraspecific comparisons involving disparate populations of Atlantic silverside (*Menidia menidia*) (Arnott et al., 2006) and rainbow trout (*Oncorhynchus mykiss*) (Claireaux et al., 2005), but it remains uncertain to what extent the relationship is found in other species.

Locomotor performance and associated metabolic costs are often coupled with life history traits, which may involve trade-offs related to growth and *M*O_2stand_ (Arnott et al., 2006; Rouleau et al., 2010). Recently, it was suggested that a trade-off between maximum sustained swimming speed (*U*_sus_) and minimum cost of transport (COT_min_) may be driving morphological diversity in axial swimmers including teleosts and cetaceans (Tokić and Yue, 2012). The trade-off assumes constraints in optimizing both *U*_sus_ and COT_min_ and suggests that aquatic species optimize either *U*_sus_ or COT_min_. Tokić and Yue (2012) applied the trade-off to models of morphological variation and reported congruent morphological variation in a number of extant aquatic species. While the trade-off may explain interspecific morphological variation, the trade-off has not been examined empirically at the intraspecific level. Likewise, it is not known if intraspecific diversity in *U*_sus_ is a source of variation in optimum swimming speed (*U*_opt_), i.e., the speed that minimizes energy expenditure per unit of distance traveled.

Using gilthead sea bream (*Sparus aurata*), *E. lateralis* and Trinidadian guppy (*Poecilia reticulata*), we employed swimming respirometry and video analyses to test four hypotheses: (1) burst activity is an indicator of anaerobic power production and correlates positively with the presence and magnitude of EPOC; (2) intraspecific diversity in MS or anaerobic capacity correlates positively with swimming performance; (3) there is a trade-off between *U*_sus_ and COT_min_ such that a high value of *U*_sus_ is associated with a high value of COT_min_ at the intraspecific level, and (4) variation in *U*_sus_ correlates positively with *U*_opt_. Data on *S. aurata* were collected for the present study, whereas data on *E. lateralis* and *P. reticulata* were derived from previous studies (Svendsen et al., 2010, 2013).

## Materials and methods

### Animals

A total of 13 gilthead sea bream (*Sparus aurata*) (body mass: 79.77 ± 2.38 g: standard length: 14.79 ± 0.24 cm (mean ± SE)) were obtained from a fish farm (Ferme Marine de Douhet) in France and kept in a flow-through holding tank (0.7 m^3^) with saltwater (30%) at 10 ± 1°C at the University of Copenhagen in Denmark. *S. aurata* were fed daily with commercial trout pellets (Biomar, Brande, Denmark). All methods applied in the present study were in agreement with current Danish regulations for the treatment and welfare of experimental animals. No fish were used more than once, and there was no mortality during any of the tests.

### Respirometry

A swimming respirometer (8.24 L) was used to measure oxygen consumption rate (*M*O_2_; mg O_2_ kg^−1^ h^−1^) as a function of swimming speed (*U*). Water temperature inside the respirometer was maintained at 10.0°C (range: 9.9–10.1°C) using a temperature controlling instrument (TMP-REG; Loligo Systems; Tjele, Denmark). The respirometer was submerged in an ambient tank supplying water for the respirometer. Air stones maintained oxygen levels >95% air saturation in the ambient tank, and the water was recirculated through a loop consisting of a separate biological filter and a UV sterilizer (model UV-1000; Tetra Pond, Melle, Germany).

The swimming section of the respirometer was 32 × 9 × 11 cm (L × W × H). An impeller placed downstream of the swimming section was driven by an external electric motor that generated a re-circulating flow. Deflectors situated upstream of the swimming section collimated the flow. To promote rectilinear flow and a uniform velocity profile in the swimming section, water passed through an upstream honeycomb (7 mm cell diameter; Plascore Inc., Michigan, USA) producing a micro turbulent flow. A grid (10 mm) in the downstream direction bounded the swimming section. A vane wheel flow sensor (Höntzsch GmbH, Waiblingen, Germany) was used to measure water speeds in the swimming section. The measurements were used for a linear correlation between water speed and voltage output from the external motor controller.

Oxygen partial pressure (kPa) in the respirometer was measured using fiber optic sensor technology (PreSens, Regensburg, Germany). Intermittent flow respirometry was applied in accordance with previous studies (Steffensen, 1989). A computer-actuated pump was employed to replace water in the respirometer through a chimney as described previously (Svendsen et al., 2013). The software AutoResp (Loligo Systems Aps, Tjele, Denmark) was used to control the flush (240 s), wait (120 s) and measurement (540 s) phases. The settings provided one measurement of *M*O_2_ per 15 min. The declining oxygen partial pressure (kPa) during the measurement phase was used to calculate *M*O_2_ (mg O_2_ kg^−1^ h^−1^) using the equation:

(1) $$MO_{2}=\frac{K\;V\;\beta }{M}$$

where *K* is the linear rate of decline (kPa h^−1^) in the oxygen content over time (h) in the respirometer, *V* is the volume of the respirometer (L) corrected for the volume of fish, β is the solubility of oxygen in the water (mg O_2_ L^−1^ kPa^−1^) (β = 0.4480) and *M* is the body mass of the fish (kg).

Preliminary trials demonstrated that the variation explained (*R*^2^) by the linear equation fitted to the declining oxygen content (kPa h^−1^), associated with each *M*O_2_ measurement, was always ≥0.95, similar to previous studies (Claireaux et al., 2006; Svendsen et al., 2012). The oxygen content never fell below 17.6 kPa. Levels of background respiration (i.e., microbial respiration) were estimated from blank runs and used to correct *M*O_2_ measurements (Jones et al., 2007; Svendsen et al., 2014).

### Burst swimming

Individual fish in the swimming section were recorded dorsally using a Hitachi video camera (model VM-H630E; Düsseldorf, Germany), situated above the swimming respirometer. A Pinnacle frame grabber (model PCTV USB2; Corel Corporation, Ontario, Canada) continuously transferred recordings to a PC, and fish 2D position (x, y coordinates) was tracked at 25 Hz using the software LoliTrack (Loligo Systems, Tjele, Denmark). A burst was defined as a forward excursion (≥4 cm) with the swimming speed increasing ≥5 cm s^−1^. The number of bursts was determined over 3 min per respirometric loop (each 15 min) and used to estimate the total number of bursts per swimming speed (each 30 min; see below).

### Experimental protocol

*S. aurata* for experiments were fasted for 48 h prior to respirometry to ensure a post-absorptive state. Fish mass (to nearest 0.01 g), length, depth and width (all to nearest 1 mm) were measured for pre-experimental calculation and correction of the solid blocking effects (Bell and Terhune, 1970; Gehrkel et al., 1990). Fish were acclimated to the respirometer for 12 h (overnight) while swimming at 0.5 body lengths per second (BL s^−1^) prior to collection of data.

After the acclimation period, routine *M*O_2_ (*M*O_2routine_) was estimated as the average *M*O_2_ during eight consecutive respirometric loops (i.e., 2 h) for each individual *S. aurata* swimming at 0.5 BL s^−1^ (i.e., acclimation speed) (Svendsen et al., 2010). At the individual level, the standard deviation (SD) of *M*O_2routine_ was calculated using the eight *M*O_2_ measurements. Next, *S. aurata* were exposed to progressive increments in the swimming speed of 0.5 BL s^−1^ every 30 min up to 2 BL s^−1^. Using 30 min intervals for each swimming speed is a common approach (Schurmann and Steffensen, 1997; McKenzie et al., 2003, 2004; Lurman et al., 2007). Two measures of *M*O_2_ were collected at each swimming speed. After completing measurements at 2 BL s^−1^, *S. aurata* were exposed to speed increments of 0.25 BL s^−1^ every 30 min.

To examine the presence and magnitude of EPOC, the swimming speed was reduced to 0.5 BL s^−1^ (acclimation speed) after each exercise level from 2 BL s^−1^ and onwards. Specifically, detection of EPOC was carried out by comparing individual *M*O_2routine_ + SD with the first post exercise *M*O_2_ measurement during the 0.5 BL s^−1^ period that followed each new swimming exercise (Svendsen et al., 2010). It was considered evidence of EPOC if the first post exercise *M*O_2_ was above *M*O_2routine_ + SD. The measurements of *M*O_2_ at 0.5 BL s^−1^ were continued until the *M*O_2_ was below *M*O_2routine_ + SD. When the *M*O_2_ stabilized below *M*O_2routine_ + SD, the swimming speed was increased to the next exercise level (i.e., the previous exercise speed + 0.25 BL s^−1^). The protocol involving incrementally increasing swimming speeds followed by the procedure to detect EPOC was continued until fatigue.

### Data acquisition and analysis

Exercise *M*O_2_ was recorded at increasing speeds from 0.5 BL s^−1^ to fatigue. Exercise *M*O_2_ as a function of *U* in individual fish was described by the exponential equation:

(2) $$MO_{2}=a\;\exp\;(Ub)$$

where *a* is the *M*O_2_ at zero speed (*U* = 0) and *b* is the rate of increase in the *M*O_2_ as a function of *U*. The intercept with the y-axis (*a*) provides an estimate of the standard metabolic rate (*M*O_2stand_) (Brett, 1964; Arnott et al., 2006; Svendsen et al., 2013). The analyses included a comparable data set on *P. reticulata* from an earlier study (Svendsen et al., 2013) in addition to the collected data on *S. aurata*. Following Svendsen et al. (2013), model fittings were limited to swim speeds without burst-assisted swimming. The analysis disregarded the measurements of post exercise *M*O_2_ at 0.5 BL s^−1^ that were inserted to evaluate EPOC after swimming speeds ≥2 BL s^−1^. Equation (2) was fitted to the individual data sets using mixed-effect models to account for temporal autocorrelation due to the repeated measurements. The analysis included an AR1 (autoregressive of order 1) covariance structure.

Maximum sustained (or aerobic) metabolic rate (*M*O_2sus_) is defined as the maximum metabolic rate that can be maintained aerobically without the accumulation of anaerobic metabolic products that contribute to fatigue and negatively impact endurance (Hillman et al., 2014). In the present study, EPOC was detected when post exercise *M*O_2_ was above *M*O_2routine_ + SD, indicating anaerobic metabolism. At the individual level, *M*O_2sus_ was measured as the maximum recorded metabolic rate (over 0.5 h) at increasing swimming speeds without evidence of EPOC. The concurrent swimming speed was used as an estimate of the maximum sustained swimming speed (*U*_sus_).

Active metabolic rate (*M*O_2active_) was defined as the maximum exercise *M*O_2_ that *S. aurata* maintained for 0.5 h without fatigue (Schurmann and Steffensen, 1997; Claireaux et al., 2005). Maximum metabolic rate (*M*O_2max_) was defined as the highest exercise *M*O_2_ measured during the complete swimming protocol (McKenzie et al., 2003; Svendsen et al., 2013; Binning et al., 2014). *M*O_2active_ and *M*O_2max_ may be different, because *M*O_2active_ is measured over 30 min, whereas *M*O_2max_ is often measured over a shorter period of time (minimum 15 min; one respirometric loop) and at a higher swim speed.

*M*O_2active_ is usually assumed to be the maximum aerobic metabolic rate (Schurmann and Steffensen, 1997); however, to what extent *M*O_2active_ includes an anaerobic component remains uncertain. If *M*O_2active_ is the maximum aerobic metabolic rate, *M*O_2active_ should not differ significantly from *M*O_2sus_. To clarify differences between metabolic rates, a one way repeated measure ANOVA was used to compare *M*O_2stand_, *M*O_2sus_, *M*O_2active_, and *M*O_2max_. The test was followed by all pairwise comparison procedures (Holm-Šídák). The same test was employed to compare the swimming speeds associated with *M*O_2sus_, *M*O_2active_, and *M*O_2max_ (i.e., *U*_sus_, *U*_active_, and *U*_max_).

The method described by Brett (1964) was used to calculate the critical swimming speed (*U*_crit_). The protocol provides measurements that are repeatable in individual fish, suggesting that *U*_crit_ represent a measure of performance, which is a lasting characteristic of the organism (Claireaux et al., 2007; Oufiero and Garland, 2009).

The magnitude of EPOC (mg O_2_ kg^−1^) was quantified using protocols published previously (Svendsen et al., 2010). When EPOC was detected, the individual relationship between time *t* (h) and post exercise *M*O_2_ was described using a double exponential equation:

(3) $$MO_{2}=\;a\exp\left(bt\right)+\;c\exp\left(dt\right)+\;MO_{2routine}$$

where *a*, *b*, *c*, and *d* are constants estimated using non-linear regression. Data included the exercise *M*O_2_ at *t* = 0. The recovery period was terminated when the fitted curve intercepted *M*O_2routine_ + SD and provided an estimate of recovery time (h). EPOC magnitude was calculated as the integrated area between the fitted curve (Equation 3) and *M*O_2routine_ from *t* = 0 to the end of the recovery period. At the individual level, EPOC was combined with the exercise *M*O_2_ to provide an estimate of the total cost of swimming (total *M*O_2_; mg O_2_ kg^−1^ h^−1^), covering both aerobic and anaerobic components. The anaerobic capacity was estimated as the maximum EPOC observed in individual fish. Anaerobic capacity was quantified as mg O_2_ kg^−1^ and mg O_2_ kg^−1^ h^−1^.

To test if the onset of burst swimming is a reliable predictor of the onset of EPOC, the minimum speed with burst swimming was correlated with the minimum speed with EPOC. The analysis was carried out using linear least square regression.

Linear mixed effects models were used to examine the relationship between the number of bursts and the magnitude of EPOC (mg O_2_ kg^−1^). Models included swimming speed as a covariate and interaction terms for swimming speed, burst number and fish identity. Temporal autocorrelation due to repeated measures was accounted for by including an AR1 covariance structure. The analysis included a comparable data set on *E. lateralis* from an earlier study (Svendsen et al., 2010).

The metabolic scope was calculated as *M*O_2max_–*M*O_2stand_ in individual fish. The hypothesis that swimming performance (*U*_crit_) is correlated with metabolic scope or anaerobic capacity in individual fish was tested using linear least square regression.

Cost of transport (COT) was calculated as mg O_2_ kg^−1^ m^−1^ using the equation:

(4) $$COT\;=\;\frac{MO_{2}}{U}$$

where *M*O_2_ is the metabolic rate (mg O_2_ kg^−1^ h^−1^), and *U* is the corresponding swimming speed (m h^−1^). The relationship between swimming speed and COT is usually U or 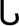 shaped with high COT values at low and high swimming speeds (Rouleau et al., 2010).

For each individual fish, COT_min_ was measured using two different approaches: (A) COT_min_ was estimated as the lowest recorded value of COT. Following this approach, the optimum swimming speed (*U*_opt_; the speed that minimizes energy expenditure per unit of distance traveled) was estimated as the swimming speed that corresponded to COT_min_; (B) COT_min_ was estimated by first determining *U*_opt_ using the equation:

(5) $$U_{opt}\;=\;\frac{1}{b}$$

where *b* originates from Equation (2) describing the individual relationship between swimming speed (cm s^−1^) and *M*O_2_ (mg O_2_ kg^−1^ h^−1^). Next, *M*O_2_ at *U*_opt_ was calculated using Equation (2); and then COT_min_ was derived using Equation (4). Results from both approaches (A and B) to estimate COT_min_ and *U*_opt_ are reported, but figures are based on approach A. The analyses included a comparable data set on *P. reticulata* from an earlier study (Svendsen et al., 2013).

In a modeling study, Tokić and Yue (2012) presented evidence for a trade-off between *U*_sus_ and COT_min_. The trade-off predicts a positive correlation between *U*_sus_ and COT_min_, i.e., superior sustained swimming performance is associated with inferior swimming economy. To examine the trade-off in *S. aurata*, individual measures of *U*_sus_ and COT_min_ were correlated using linear least square regression. Similarly, this study tested for a relationship between *U*_sus_ and *U*_opt_ in individual fish. In addition to the data on *S. aurata*, the analyses of *U*_sus_, COT_min_, and *U*_opt_ included a comparable data set derived from an earlier study on *P. reticulata* (Svendsen et al., 2013).

Data were transformed [e.g., ln(*x* + 1)] to meet the normality and homoscedasticity requirements of parametric analyses. The free statistical software R (R Development Core Team, 2014) and SigmaPlot (Systat Software, Erkrath, Germany) were used for statistical analyses and graphing. The R package nlme (Pinheiro et al., 2011) was employed to fit models. Results were considered significant at *P* < 0.05. All values are reported as means ± SE unless otherwise noted.

## Results

### Metabolic rates and swimming performance

**Figure 1:**
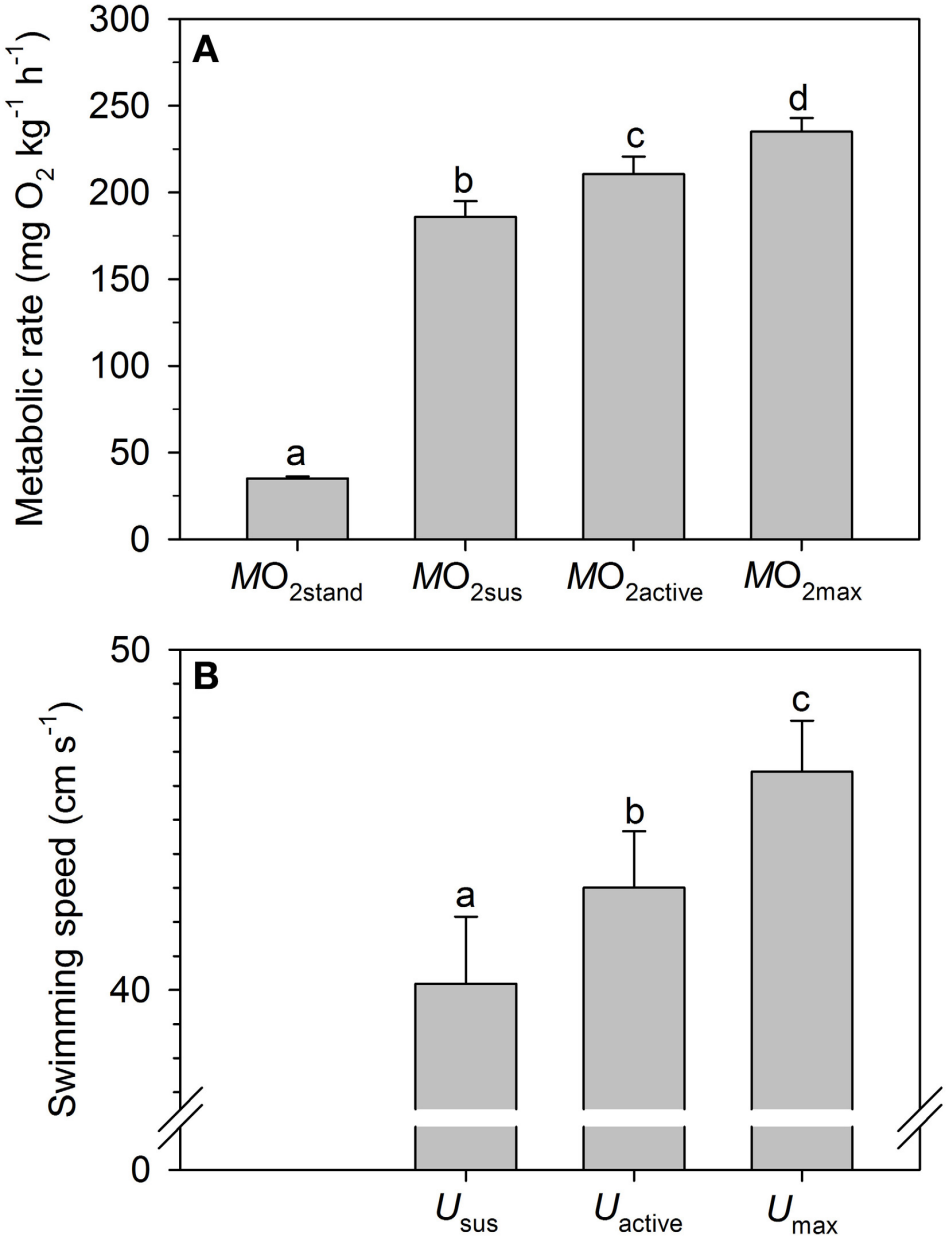
**Measurements of (A) metabolic rate (mg O_2_ kg^−1^ h^−1^) and (B) swimming speed (cm s^−1^) in gilthead sea bream (*Sparus aurata*)**. Data include standard metabolic rate (*M*O_2stand_), maximum sustained (or aerobic) metabolic rate (*M*O_2sus_), active metabolic rate (*M*O_2active_) and maximum metabolic rate (*M*O_2max_). The measurements are defined in detail in the text. Corresponding swimming speeds **(B)** were derived from the metabolic measurements and include maximum sustained (*U*_sus_), active (*U*_active_) and maximum (*U*_max_) swimming speeds. There are significant differences (*P* < 0.05) between the measurements of metabolism and swimming speed as indicated by the different letters.

*M*O_2stand_, *M*O_2sus_, *M*O_2active_, and *M*O_2max_ were measured at increasing speeds (Figure 1) and were all statistically different (*P* < 0.05). Notably, *M*O_2sus_ was lower than *M*O_2active_, providing evidence of anaerobic metabolism (EPOC) in a significant number of *S. aurata* exercising at the level of *M*O_2active_ (Figure 1A). The finding suggests that *M*O_2sus_ is a more appropriate measure of maximum sustained (or aerobic) metabolic rate than *M*O_2active_. Similar to the metabolic values, the corresponding swimming speeds (*U*_sus_, *U*_active_, and *U*_max_) differed significantly (*P* < 0.05) (Figure 1B). Interestingly, *U*_sus_ varied twofold between individuals with measurements ranging between 27 and 53.2 cm s^−1^. Measures of *U*_crit_ were not included in Figure 1, but ranged between 35.3 and 56.5 cm s^−1^, with an average value of 45.0 ± 1.6 cm s^−1^. *M*O_2sus_ and *U*_sus_ corresponded to 79.3 ± 3.3% of *M*O_2max_ and 88.9 ± 1.9% of *U*_crit_, respectively, with anaerobic metabolism detected above these exercise levels.

### Exercise *M*O_2_ and total *M*O_2_ in relation to *U*_crit_

**Figure 2:**
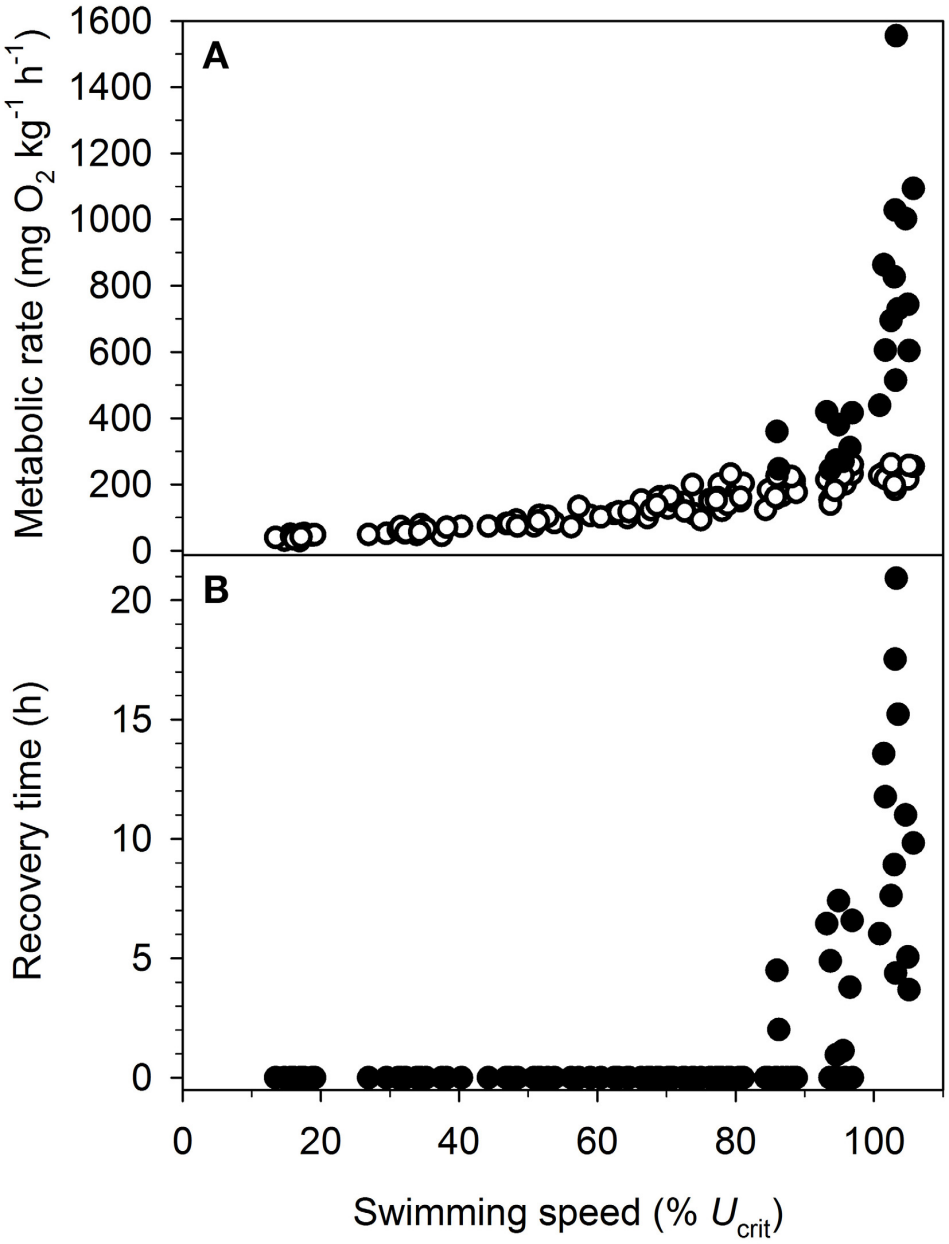
**(A)** Metabolic rate (mg O_2_ kg^−1^ h^−1^) and **(B)** recovery time (h) in relation to swimming speed (%*U*_crit_) in individual gilthead sea bream (*Sparus aurata*). *U*_crit_ is the critical swimming speed and defined in detail in the text. For **(A)**, metabolic rates were measured in the swimming fish (exercise *M*O_2_; open symbols) and as excess post exercise oxygen consumption (EPOC; mg O_2_ kg^−1^). Exercise *M*O_2_ and EPOC were combined to estimate the total metabolic cost of swimming (total *M*O_2_; closed symbols). For **(B)**, recovery time (h) reflects the duration of EPOC after single swimming exercises (up to 30 min).

EPOC was detected at all swimming speeds faster than *U*_sus_ and was combined with the exercise *M*O_2_ to estimate the total *M*O_2_. Because of the observed intraspecific variation in swimming performance, exercise *M*O_2_ and total *M*O_2_ were plotted as a function of %*U*_crit_ (Figure 2A) similar to previous studies (Lurman et al., 2007; Tudorache et al., 2008; Teulier et al., 2013). EPOC contributed to the total *M*O_2_ starting at 86% of *U*_crit_ (Figure 2A). EPOC constituted 53.5 ± 4.9% of the total *M*O_2_, ranging from 14.2 to 86.4% of total *M*O_2_, at swimming speeds with evident EPOC. Thus, EPOC frequently constituted more than half of the swimming costs. Recovery time associated with EPOC lasted 7.8 ± 1.1 h, ranging from 1.0 to 20.9 h (Figure 2B).

### Positive correlations between burst activity and anaerobic metabolism (EPOC)

**Figure 3:**
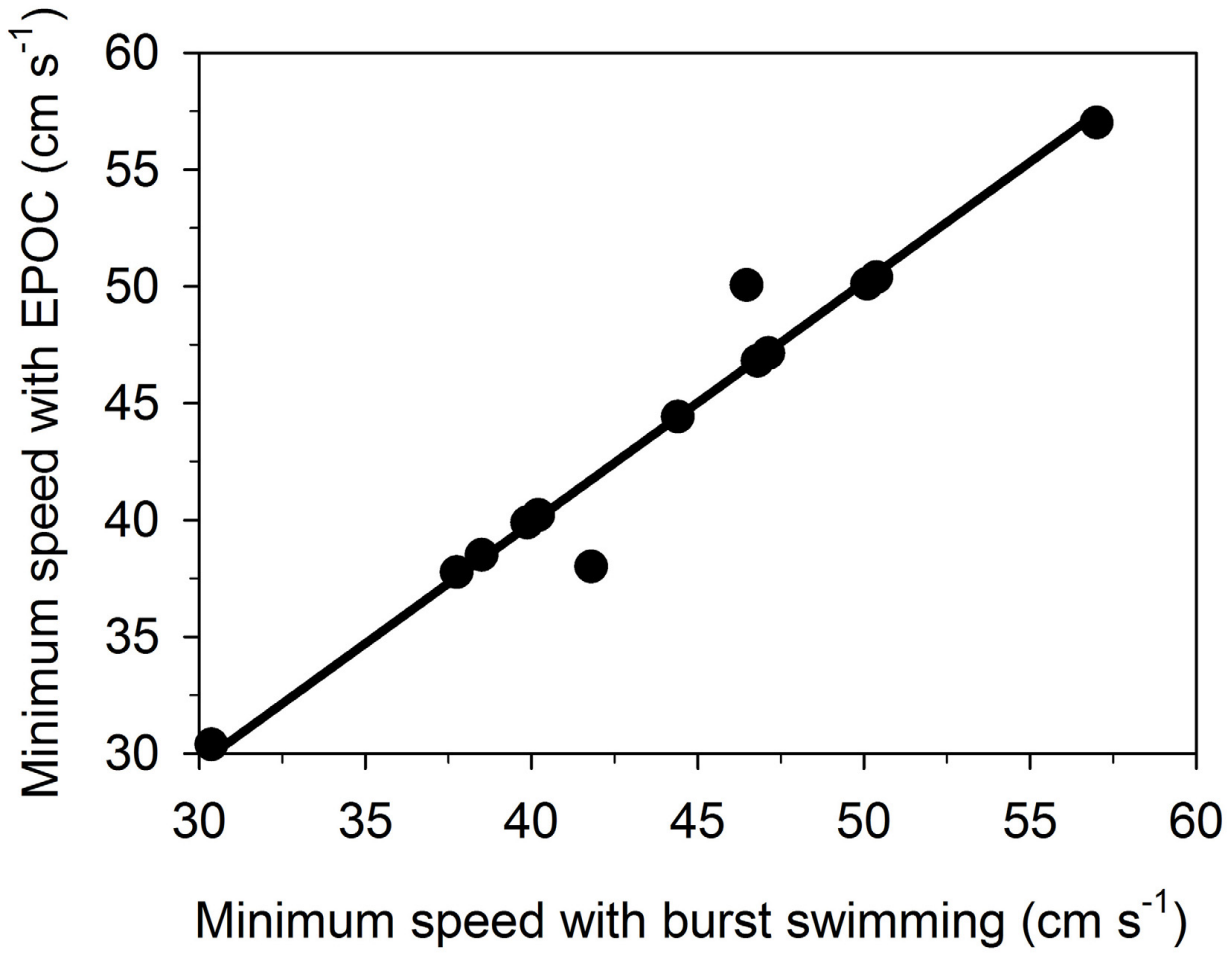
**The minimum speed with excess post exercise oxygen consumption (EPOC; mg O_2_ kg^−1^) correlates positively with the minimum speed with burst swimming (*P* < 0.0001; *R*^2^ > 0.95) in gilthead sea bream (*Sparus aurata*)**. The relationship shows that the onset of burst swimming at increasing speeds indicates the onset of EPOC and therefore, anaerobic metabolism in individual fish.

There was a positive linear relationship (*P* < 0.0001; *R*^2^ > 0.95) between the minimum speed with EPOC and the minimum speed with burst swimming (Figure 3). The intercept with the y-axis was not significantly different from zero (*P* > 0.65). The relationship shows that the onset of burst swimming is a strong predictor of the onset of EPOC and anaerobic metabolism at increasing swimming speeds.

**Figure 4:**
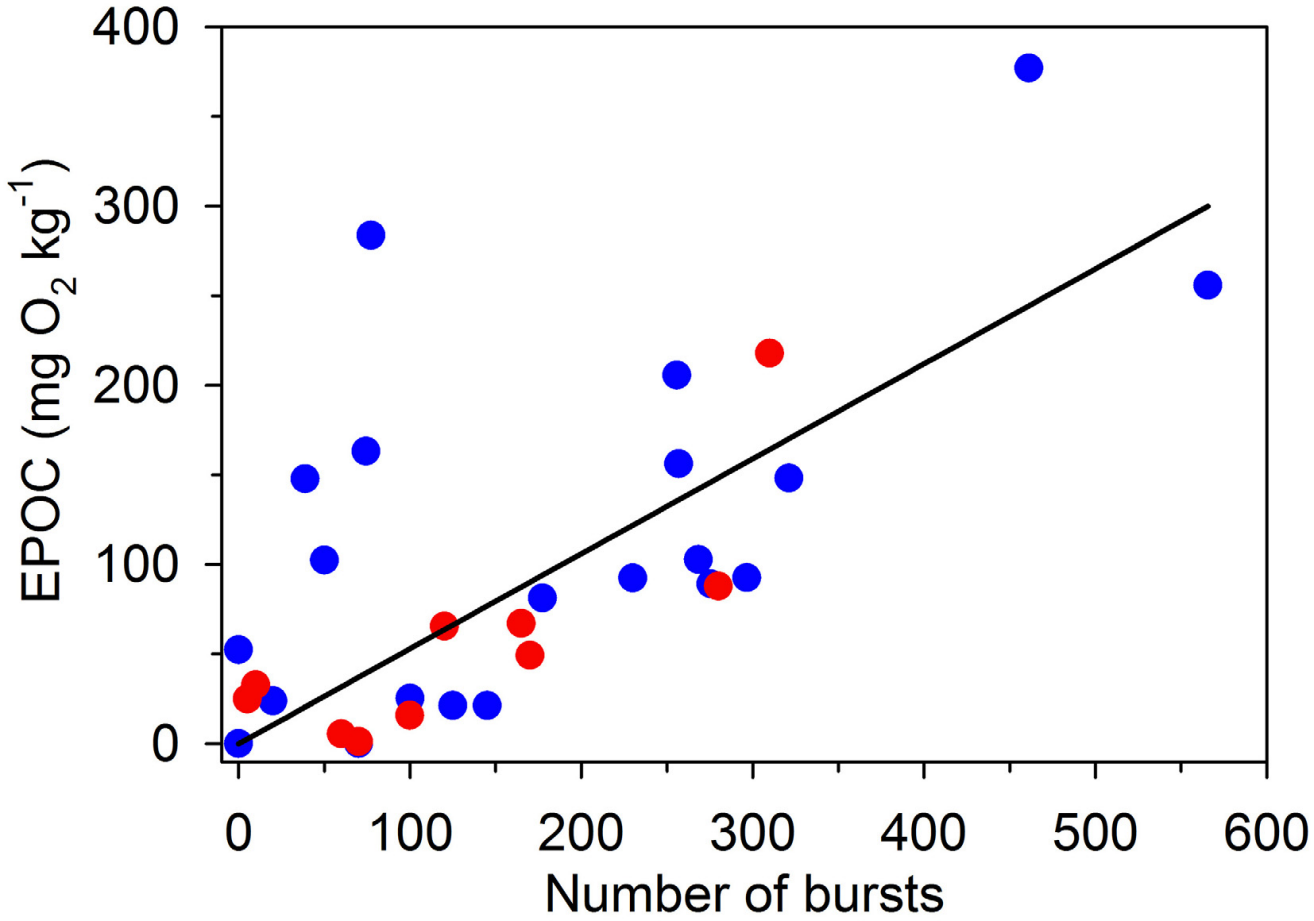
**The magnitude of excess post exercise oxygen consumption (EPOC; mg O_2_ kg^−1^) correlates positively with burst activity in gilthead sea bream (*Sparus aurata*) (blue symbols) and striped surfperch (*Embiotoca lateralis*) (red symbols)**. Data on *S. aurata* are from the present study, whereas data on *E. lateralis* are from Svendsen et al. (2010). The linear fit (*P* < 0.0001) reflects the pooled data set for both species (Equation 6), because species-specific regression slopes and intercepts with the y-axes are not statistically different (*P* > 0.34). The relationship suggests that a burst represents a metabolic cost of 0.53 mg O_2_ kg^−1^.

The relationship between the number of bursts and magnitude of EPOC was examined using a linear mixed effects model. The model included swimming speed as a covariate, but no significant effect (*P* > 0.25) or interactions (*P* > 0.64) related to swimming speed was detected. Model terms for swimming speed and interactions were therefore eliminated from further analyses. A comparable data set from a previous study on *E. lateralis* (Svendsen et al., 2010) was included in the analysis. For both data sets, the intercept with y-axis was not significantly different from zero (*P* > 0.34) and the slopes did not differ between the two data sets (*P* > 0.94). These findings indicated that the relationships between burst numbers and EPOC were similar in the two species, and the data were therefore, combined. The resulting common relationship (Figure 4) was described by the equation (*P* < 0.0001):

(6) EPOC=0.53 (±0.05) bursts

The relationship indicates that each burst corresponds to an average metabolic cost of 0.53 mg O_2_ kg^−1^ (Figure 4).

### No correlation between *U*_crit_ and metabolic scope or anaerobic capacity

Metabolic scope was estimated as *M*O_2max_–*M*O_2stand_, whereas anaerobic capacity was estimated as the maximum EPOC observed in individual fish. The maximum EPOC value was always associated with fish fatigue. There was no evidence that individual *U*_crit_ correlated with metabolic scope (*P* > 0.87; *R*^2^ < 0.01) or with anaerobic capacity (*P* > 0.57; *R*^2^ < 0.04) (data not shown). The analyses of anaerobic capacity involved maximum EPOC quantified as mg O_2_ kg^−1^ and mg O_2_ kg^−1^ h^−1^.

### No trade-off between *U*_sus_ and COT_min_

**Figure 5:**
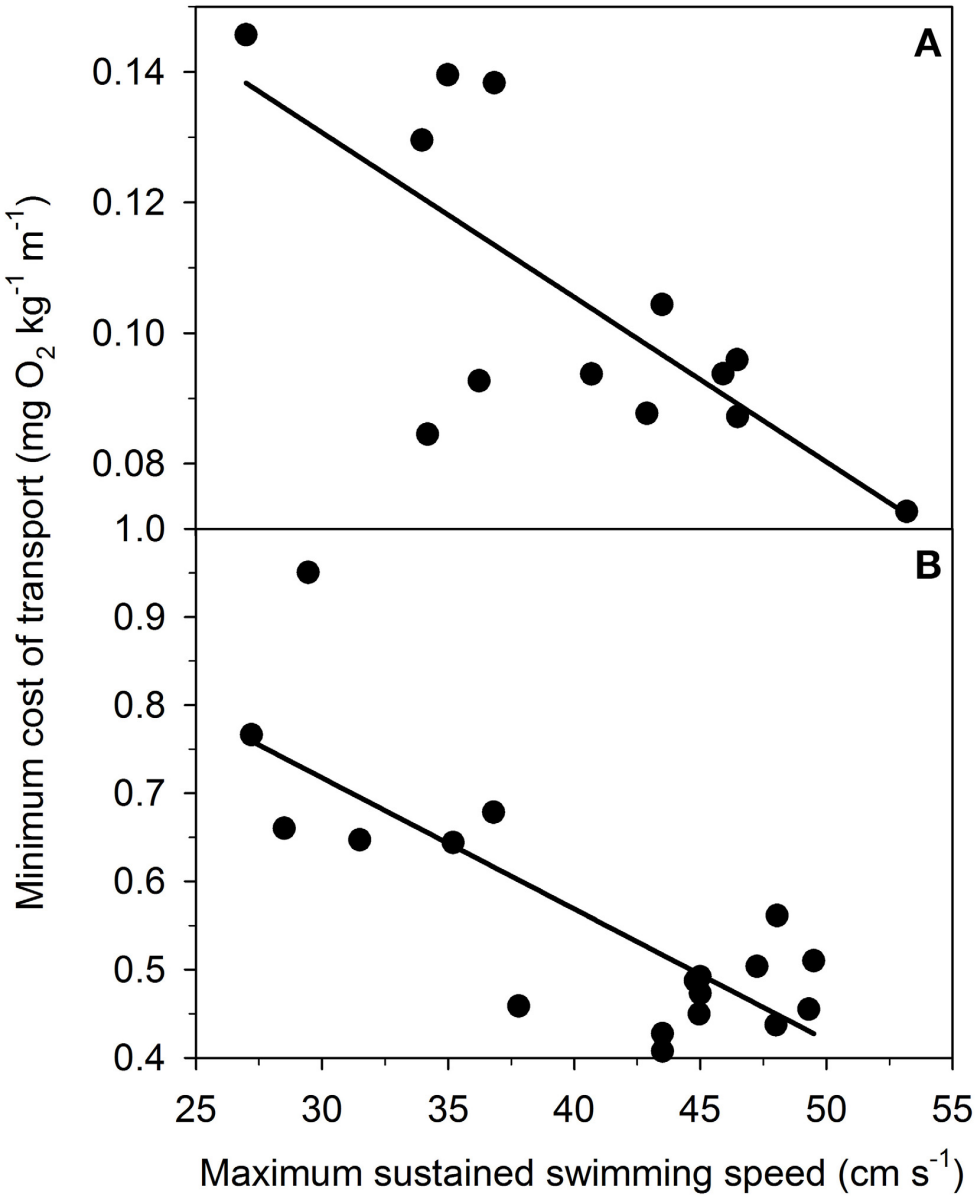
**Minimum cost of transport (COT_min_; mg O_2_ kg^−1^ m^−1^) correlates negatively with maximum sustained swimming speed (*U*_sus_; cm s^−1^) in (A) gilthead sea bream (*Sparus aurata*; *n* = 13) and (B) Trinidadian guppy (*Poecilia reticulata*; *n* = 18)**. Data on *S. aurata* are from the present study, whereas data on *P. reticulata* are derived from Svendsen et al. (2013). Both relationships are statistically significant (*P* < 0.005; *R*^2^ > 0.53). The relationships suggest that superior sustained swimming performance (i.e., high *U*_sus_) is associated with superior swimming economy (i.e., low COT_min_) in both species. Note that y-axes differ between the two panels.

This study examined a trade-off between *U*_sus_ and COT_min_ by comparing swimming performance and metabolism in *S. aurata* and *P. reticulata.* In terms of *S. aurata*, *U*_sus_ was assumed to correspond to the highest swimming speed without EPOC (Figures 1, 2). Data on *P. reticulata* were derived from Svendsen et al. (2013). While EPOC was not measured in *P. reticulata*, the study quantified burst activity in individual *P. reticulata* at increasing speeds. Using the relationship between the onset of burst swimming and the onset of EPOC (Figure 3), EPOC occurrence at increasing speeds, and thereby *U*_sus_, were estimated in individual *P. reticulata*. COT_min_ in *P. reticulata* was estimated in the same fashions (approaches A and B) as in *S. aurata* (Equations 4 and 5). The relationships between *U*_sus_ and COT_min_ were examined using linear least square regressions (Figure 5). For both species, there was no evidence of a trade-off between *U*_sus_ and COT_min_. In fact, there were significant negative correlations between *U*_sus_ and COT_min_, revealing that individuals exhibiting superior sustained swimming performance (i.e., high *U*_sus_) also exhibit superior swimming economy (i.e., low COT_min_) (Figure 5). The negative correlations between *U*_sus_ and COT_min_ were evident in both species and regardless of the approach (A and B) used to estimate COT_min_ (all *P* < 0.005; *R*^2^ > 0.53). Data in Figure 5 are based on approach A.

### Positive correlations between *U*_sus_ and *U*_opt_

**Figure 6:**
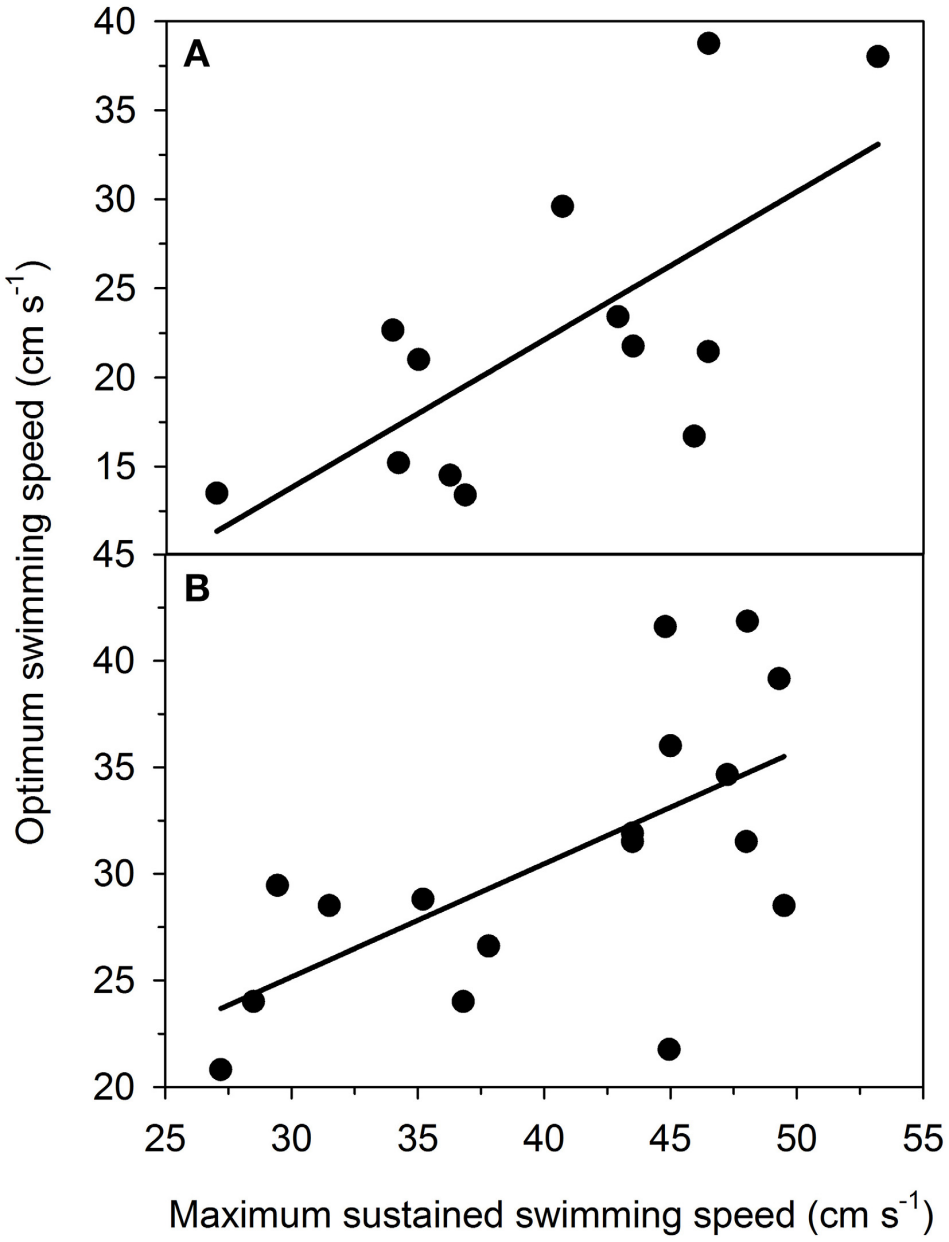
**Optimum swimming speed (*U*_opt_; cm s^−1^) correlates positively with maximum sustained swimming speed (*U*_sus_; cm s^−1^) in (A) gilthead sea bream (*Sparus aurata*; *n* = 13) and (B) Trinidadian guppy (*Poecilia reticulata*; *n* = 18)**. Data on *S. aurata* are from the present study, whereas data on *P. reticulata* are derived from Svendsen et al. (2013). Both relationships are statistically significant (*P* < 0.005; *R*^2^ > 0.40). The relationships suggest that superior sustained swimming performance (i.e., high *U*_sus_) is associated with superior optimum swimming speed (i.e., high *U*_opt_) in both species. Note that y-axes differ between the two panels.

There were significant positive correlations between *U*_sus_ and *U*_opt_ (Figure 6). The analyses included data on *S. aurata* (Figure 6A) and *P. reticulata* (Figure 6B) and revealed that individuals exhibiting superior sustained swimming performance (i.e., high *U*_sus_) also exhibit superior optimum swim speed (i.e., high *U*_opt_). The positive correlations between *U*_sus_ and *U*_opt_ were evident in both species and regardless of the approach used to estimate *U*_opt_ (approach A: all *P* < 0.005; *R*^2^ > 0.40; approach B: all *P* < 0.05; *R*^2^ > 0.26). Data in Figure 6 are based on approach A.

## Discussion

This study demonstrated the energetic importance of anaerobic metabolism during unsteady locomotion. There was no evidence of *U*_crit_ correlating with MS or anaerobic capacity. Moreover, we provided intraspecific evidence that a high *U*_sus_ is coupled with low COT_min_ and high *U*_opt_ in individual fish. Specifically, our results reveal that burst swimming is associated with anaerobic metabolism and a substantial metabolic cost, which is expressed as EPOC. Our intraspecific results on two teleost species are at odds with the conjecture that there is a trade-off between *U*_sus_ and COT_min_ as indicated by Tokić and Yue (2012). By applying the trade-off, the authors provided a model that explained variation in morphology in various teleost and cetacean species. In contrast, the present study is based on intraspecific data collected empirically. Our findings suggest that intraspecific variation in *U*_sus_ and COT_min_ is not driven by a trade-off producing a high *U*_sus_ in some individuals and a low COT_min_ in other individuals. Because the results suggest that *U*_sus_ and COT_min_ are optimized concurrently, it is unlikely that the trade-off drives intraspecific morphological variation.

Previous studies have demonstrated that the *U*_crit_ protocol includes swimming powered by both aerobic and anaerobic metabolism (Burgetz et al., 1998; Richards et al., 2002). In *E. lateralis*, EPOC and anaerobic metabolism is present at 88% of *U*_crit_ (Svendsen et al., 2010). Corroborating previous results, the present study found evidence of EPOC starting at swimming speeds corresponding to 86% of *U*_crit_. At higher speeds, EPOC increased rapidly and constituted up to 86% of the total *M*O_2_. The maximum value of EPOC was always associated with fatigue. Likewise, beginning at 89% of *U*_crit_ in Atlantic cod (*Gadus morhua*), Lurman et al. (2007) found evidence of anaerobic metabolism as indicated by decreasing levels of phosphocreatine and intracellular pH and increasing levels of inorganic phosphate. Our study corroborates that the *U*_crit_ protocol involves depletion of both aerobic and anaerobic resources, and shows that the metabolic costs associated with the recovery from the anaerobic perturbation (i.e., EPOC) may constitute the majority of the swimming costs. The results highlight the importance of measuring both exercise *M*O_2_ and EPOC to estimate the total metabolic costs of swimming in fish approaching prolonged and burst swimming speeds. In the absence of EPOC measurements, the metabolic cost of swimming may be significantly underestimated.

This study shows that the onset of burst-assisted swimming is closely related to the onset of EPOC at increasing swimming speeds in individual fish. The initiation of burst swimming is therefore a strong predictor of EPOC and anaerobic metabolism. Similarly, we found that the magnitude of EPOC increases linearly with the number of bursts. The present data are consistent with a previous study on *E. lateralis* (Svendsen et al., 2010). Combining the two data sets suggests that each burst corresponds to an energetic cost of 0.53 mg O_2_ kg^−1^. *E. lateralis* is a labriform swimmer (i.e., pectoral fins used for propulsion at low and medium swimming speeds) whereas *S. aurata* is an axial swimmer (i.e., axial undulation used for propulsion). The fact that we found no differences in the two relationships between bursts and EPOC indicates that the metabolic cost of burst swimming may be similar across fish species employing disparate types of locomotion.

MS is predicted to play a major role in relation to effects of climate change, and other anthropogenic stressors including hypoxia, on aquatic exothermic animals (Claireaux and Lefrançois, 2007; Chabot and Claireaux, 2008; Guderley and Pörtner, 2010; Pörtner, 2010; Pörtner and Peck, 2010; McBryan et al., 2013; Seth et al., 2013; Di Santo, 2015). It remains uncertain, however, to what extent intraspecific diversity in MS varies with other important physiological traits including locomotor performance. The present study measured intraspecific variation in MS and anaerobic capacity (i.e., maximal EPOC) in *S. aurata* and correlated data with individual variation in swimming performance (*U*_crit_). We found no evidence that diversity in MS or anaerobic capacity correlates with *U*_crit_ (*P* > 0.57) indicating that other factors, including morphological (Rouleau et al., 2010) or biomechanical (Svendsen et al., 2013) traits, drive the variation in swimming performance.

It is possible that the lacking relationship between MS and *U*_crit_ was caused by our method of measuring *M*O_2max_. Similar to previous studies (McKenzie et al., 2003; Svendsen et al., 2013; Binning et al., 2014), we used an *U*_crit_ protocol to measure *M*O_2max_ involving progressive increments in the swimming speed of 0.25 BL s^−1^ every 30 min, starting from 2 BL s^−1^ and until fatigue. Our protocol differed, however, from conventional protocols, because we inserted periods with swimming speeds adjusted to 0.5 BL s^−1^ (acclimation speed) for measurements of EPOC after each swimming speed ≥ 2 BL s^−1^. Although the mechanistic basis is unknown, it is possible that our protocol affected the measurements of *M*O_2max_. As an alternative to the *U*_crit_ protocol, a number of recent studies have used a chase protocol to measure *M*O_2max_ (Norin and Malte, 2011, 2012; Svendsen et al., 2014). The *U*_crit_ protocol is often assumed to provide measures of *M*O_2max_ (Farrell and Steffensen, 1987; Hammer, 1995) and may in fact elicit values of *M*O_2max_ that are higher than the values elicited by the chase protocol (Roche et al., 2013). Therefore, it is unlikely that a significant relationship between MS and swimming performance would have been revealed if we had used a chase protocol instead of the *U*_crit_ protocol to measure *M*O_2max_. In humans, *M*O_2max_ is typically measured using test protocols that are much faster (Barker et al., 2011; Vanhatalo et al., 2011; Mauger et al., 2013) than the *U*_crit_ protocol used in the present study. While a protocol that continuously steps up the swimming speed in much faster pace than the *U*_crit_ protocol might produce higher values of *M*O_2max_ (and therefore MS) and swimming performance (Farrell, 2008), it remains to be tested if the methodology would produce a significant relationship between MS and swimming performance. A faster protocol would rely more on anaerobic metabolism to power swimming (Farrell, 2008; Poulsen et al., 2012), and so a relationship between anaerobic capacity and swimming performance might be revealed.

A recent study emphasized a trade-off between *U*_sus_ and COT_min_ driving morphological diversity in aquatic locomotion (Tokić and Yue, 2012). The trade-off assumes constraints in optimizing *U*_sus_ and COT_min_ simultaneously, suggesting that aquatic species may optimize either *U*_sus_ or COT_min_. By applying the trade-off, Tokić and Yue (2012) modeled morphological variation and reported congruent morphological variation in several extant aquatic species. The present study examined the trade-off within two teleost species and found no support for the trade-off. In fact, data revealed a significant negative correlation between *U*_sus_ and COT_min_, suggesting that individuals with high *U*_sus_ also exhibit low COT_min_. The negative relationship indicates that the two traits are optimized simultaneously and could be related to the same mechanistic basis without constraints. Interestingly, studies are increasingly uncovering significant intraspecific variation in locomotor performance and metabolic rate (Nelson et al., 2003; Langerhans, 2008, 2009a; Dalziel et al., 2011, 2012; Dalziel and Schulte, 2012; Svendsen et al., 2013; Binning et al., 2014). The present study indicates that intraspecific morphological variation, associated with intraspecific variation in locomotor performance and metabolic rate, is not driven by a trade-off between *U*_sus_ and COT_min_.

There are a number of reasons why we may not observe a trade-off between *U*_sus_ and COT_min_ in our intraspecific data. Variation between species is much more pronounced than between individuals of the same species. Therefore, interspecific variation may better reflect the full spectrum of functional trade-offs that influences morphological variation related to aquatic locomotion. It is also possible that a trade-off between *U*_sus_ and COT_min_ is present in the two tested fish species, but not expressed at the whole-organism level, because of compensating or masking factors involving morphological, physiological and/or biomechanical traits. Moreover, our estimates of *U*_sus_ and COT_min_ based on respirometry and video analysis might be misleading. For example, it is possible that estimates of *U*_sus_ using measures of EPOC (*S. aurata*) and burst-assisted swimming (*P. reticulata*) do not accurately reflect maximum sustained swimming speeds. *M*O_2_ is, however, a well-established proxy for aerobic metabolic rate, and the gait transition from steady to unsteady (i.e., burst-assisted) swimming is a well-known indicator of the shift from aerobic to anaerobic power production (Peake and Farrell, 2004, 2006; Peake, 2008; Svendsen et al., 2010). Similarly, it is possible that the use of forced linear swimming to estimate *U*_sus_ and COT_min_ provides results that do not necessarily reflect natural conditions, because fish typically swim spontaneously in a non-linear fashion with the relationship between swimming speed and metabolic rate differing from linear swimming (Steinhausen et al., 2010).

Diversity in locomotor performance and metabolism can be important sources of variation in animal behaviors. For example, Hillman et al. (2014) suggested that variation in physiological capacity for movement influences dispersal and therefore fine-scale genetic structure of several vertebrate groups. At the intraspecific level, physiological performance is an important determinant of behaviors related to schooling (Killen et al., 2011), territory acquisition and defense and foraging (Breau et al., 2011; Killen et al., 2014). Likewise, physiological and energetic states may influence behaviors in migratory species (Poulsen et al., 2010; Boel et al., 2014). Recent studies have shown that exercise training that increases swimming performance may change the behavior of animals and cause elevated boldness and exploratory tendency (Sinclair et al., 2014). The mechanistic basis of the relationship between exercise training and behavior could be related to the positive relationship between *U*_sus_ and *U*_opt_ found in the present study. Because exercise training increases aerobic potentials in red and white musculature (Davison, 1997) and swimming performance (Farrell et al., 1990; Sinclair et al., 2014), exercise training should also elevate *U*_sus_ and therefore *U*_opt_. Typically, fish swim spontaneously at speeds corresponding to *U*_opt_ (Videler, 1993; Tudorache et al., 2011). This hypothesis suggests that exercise training increases spontaneous swimming speeds via the positive relationship between *U*_sus_ and *U*_opt_. It seems likely that increased spontaneous swimming speed is associated with elevated boldness and exploratory tendency as observed by Sinclair et al. (2014). Therefore, the positive relationship between *U*_sus_ and *U*_opt_ could provide a mechanistic link between physiological and behavioral phenotypes. Nevertheless, this hypothetical framework warrants additional study to clarify the mechanistic basis of intraspecific correlations between physiological and behavioral phenotypes.

## Author contributions

Conceived and designed the experiments: BT, JCS, JFS. Performed the experiments: BT. Analyzed the data: JCS, GAC, BT. Contributed reagents/materials/analysis tools: JFS. Wrote the paper: JCS. Revised the manuscript critically for important intellectual content: JCS, BT, GAC, JFS.

### Conflict of interest statement

The authors declare that the research was conducted in the absence of any commercial or financial relationships that could be construed as a potential conflict of interest.

## Abbreviations

EPOC: Excess post exercise oxygen consumption
BL: Body length
COT: Cost of transport
COT_min_: Minimum cost of transport
Exercise *M*O_2_: Metabolic rate measured in swimming fish (i.e., instantaneous metabolic rate)
*M*O_2_: Metabolic rate
*M*O_2active_: Active metabolic rate defined as the maximum metabolic rate maintained for 0.5 h
*M*O_2max_: Maximum metabolic rate defined as the maximum metabolic rate measured at increasing swimming speeds
*M*O_2routine_: Routine metabolic rate defined as the average metabolic rate in fish swimming at 0.5 BL s^−1^
*M*O_2stand_: Standard metabolic rate (i.e., *a* in Equation 2)
*M*O_2sus_: Maximum sustained metabolic rate defined as the maximum metabolic rate (over 0.5 h) without any EPOC (i.e., no influence of anaerobic metabolism)
Total *M*O_2_: Exercise *M*O_2_ and EPOC combined as an estimate of the total metabolic swimming cost
*U*_active_: Swimming speed associated with the active metabolic rate (*M*O_2active_)
*U*_crit_: Critical swimming speed
*U*_max_: Swimming speed associated with the maximum metabolic rate (*M*O_2max_)
*U*_opt_: Optimum swimming speed defined as the speed that minimizes energy expenditure per unit of distance traveled
*U*_sus_: Maximum sustained swimming speed defined as the maximum recorded swimming speed (over 0.5 h) without any EPOC (i.e., no influence of anaerobic metabolism).
